# Prediction of Crowd Behavior in Holy Sites: Psychological, Social, and Risk Perception Factors among Pilgrims 

**DOI:** 10.12688/f1000research.180247.2

**Published:** 2026-07-22

**Authors:** Motaz Alotaibi, Yahya Khatatbeh

**Affiliations:** 1 Department of Psychology, College of Social Science, Imam Mohammad Ibn Saud Islamic University (IMSIU), Riyadh, Saudi Arabia

**Keywords:** Crowd behavior, risk perception, psychological factors, social factors, Hajj, crowd management

## Abstract

**Background:**

Large religious gatherings such as Hajj involve complex psychological and social processes that influence pilgrims’ experiences and crowd behavior. This study examined risk perception, psychosocial factors, and adaptive crowd behavior among pilgrims, explored the relationships among these variables, and evaluated their predictive contribution to crowd behavior.

**Methods:**

A descriptive, correlational, and predictive cross-sectional design was employed. A purposive sample of 407 pilgrims completed validated measures of risk perception, psychosocial factors, and crowd behavior. Descriptive statistics, univariate between-subjects analyses, Pearson’s correlation coefficients, and multiple regression analyses were performed.

**Results:**

Pilgrims demonstrated moderate-to-high levels of risk perception and adaptive crowd behavior, together with balanced psychosocial characteristics. Nationality was significantly associated with risk perception, psychosocial factors, and crowd behavior (all p < 0.001), whereas economic level was associated only with psychosocial factors (p = 0.041). No significant differences were observed according to age or educational level. Positive correlations were found among the principal study variables. The regression model was statistically significant (R ^2^ = 0.181, p < 0.001). Psychosocial factors significantly predicted adaptive crowd behavior (β = 0.391, p < 0.001), whereas risk perception was not a significant independent predictor after psychosocial factors were entered into the model (β = 0.049, p = 0.413).

**Conclusion:**

Risk perception was associated with adaptive crowd behavior at the bivariate level, whereas psychosocial factors emerged as the significant independent predictor in the regression model. Strengthening institutional trust, personal readiness, learning, and spiritually relevant communication may support safer and more cooperative crowd behavior during Hajj. Integrating psychosocial considerations into crowd-management and risk-communication strategies may enhance safety and well-being during major religious gatherings.

## Introduction

Millions of Muslims worship in Makkah during Hajj, one of the world’s most difficult mass gatherings. Psychosocial variables impact the crowd behavior and individual well-being of large religious events, and shared social identity predicts cooperation better than crowd density/location ( Alnabulsi & Drury, 2014). A study on Hajj pilgrims has highlighted the interplay between crowd management, cultural diversity, and psychological readiness ( Abd Rahman et al., 2019). No matter what their value is, mass meetings seem to boost a sense of belonging in the community, as well as social cohesiveness and personal efficacy through emotional synchrony ( Páez et al., 2015). When it comes to Indian pilgrims, there’s this common pattern where stress-related psychological problems show up during mass gatherings, and a noticeable share of those pilgrims need social support or even clinical help ( Khan et al., 2016). The Hajj brings in millions of Muslims, and it’s not just numbers; religious identity, emotional synchrony, and community norms shape how the crowd behaves. Also, the interpretation of threats is influenced by lived experience, trust in the regulators, group identification, and religion ( Samarkandi et al., 2025). Additionally, religious commitment and shared group identity are associated with reduced fear responses in collective settings ( Alnabulsi & Drury, 2014). Indeed, reassurance can quiet crowds, and a closed-knit community based on religion and risk-awareness education makes individuals more disciplined and helpful and less likely to join stampedes and disorder movements. According to social identity theory and identity fusion theory, group engagement fosters loyalty, solidarity, and collaboration ( Durkheim, 2016; Páez et al., 2015; Swann Jr et al., 2009; Wlodarczyk et al., 2020). Durkheim’s (2016) collective effervescence defines how the emotional synchrony of rituals binds respondents and reinforces symbols and social norms. Other studies also describe that group identity and shared traditions encourage members to follow community norms and expectations, thereby supporting cooperation and social order within collective religious contexts ( Drury & Reicher, 2000). Large meetings boost mental health, social integration, and group performance, despite them also producing congestion or being characterized by limited resources ( Wlodarczyk et al., 2020). These psychosocial benefits may support collective worship in holy locations. In terms of scale, such gatherings can be well illustrated by the 2013 Kumbh Mela in India, which attracted an estimated 61 million respondents over its duration and approximately 25 million on its peak day ( Khan et al., 2016). Therefore, the literature indicates that shared social group membership among large religious event respondents fosters a sense of security and belonging.

The science of crowds has extensively studied crowd behavior at mass gatherings. The groundwork was laid by Fruin (1993) who looked at the impact of crowd density and movement patterns on crowd safety in terms of the Level of Service. Also, Helbing et al. (2000) proposed the Social Force Model, showing that the interaction of individual pedestrians with the constraints of the environment can generate collective crowd phenomena such as congestion and crowd turbulence. Still (2014) emphasized the importance of crowd risk analysis by extending crowd safety assessment with physical density, movement dynamics, and situational risk factors. These methods have significantly enhanced the understanding of crowd movement and management in high-density environments. Nevertheless, their attention has largely been directed towards physical, engineering and operational determinants of crowd behavior. Relativelyless attention has been paid to psychosocial mechanisms that influence behavioral adaptation in religious mass gatherings, such as the Hajj pilgrimage.

Following these insights from crowd science, recent research has more and more pointed out that crowd behaviour cannot be fully explained just in terms of physical and environmental conditions. At the same time, psychological processes, social identity, spirituality, institutional trust and risk perception seem to matter too, like a lot. People also try to make sense of a crowd situation through those inner and social lenses, and then they respond in a different way, especially when we’re talking about religious pilgrimages ( Alnabulsi & Drury, 2014; Páez et al., 2015; Wlodarczyk et al., 2020). Risk perception is a key construct in understanding human responses to potentially hazardous situations. In Slovic’s psychometric paradigm, people do not evaluate risks based purely on objective probabilities, but rely on subjective judgments that are influenced by perceived severity, familiarity, controllability, trust, and emotional reactions. In the context of mass gatherings, perceived risk determines how individuals interpret crowd-related threats and their preparedness, decision-making and behavioral responses. Hence risk perception is a multi-dimensional psychological process that goes beyond the simple awareness of risk and provides an important framework for understanding adaptive behavior during large-scale religious gatherings such as the Hajj pilgrimage ( Slovic, 1987).

There are many recent studies which delved into crowd behavior at major religious events, most being descriptive and context-specific. This is despite social identity, spirituality, educational readiness, and prosocial conduct impacting crowd behavior in general instead of just those behaviors in specific contexts. Moreover, few studies have used integrative or predictive models to analyze how these components produce adaptive and non-adaptive crowd behaviors. For example, Clingingsmith et al. (2009) evaluated 1,605 Muslim pilgrims and Pakistani religion and culture after the Hajj pilgrimage, showcasing that the experience was characterized by tolerance and disregard for local standards. In this cited study, Turkish clinicians also assessed 294 Hajj pilgrims seeking mental health treatments, along with their demographics and socioeconomics (59% women, 41% men; average age, 53 years). The clinicians used DSM-IV criteria to diagnose depression (26.5%), anxiety disorders (49%), major psychotic illnesses, dementia, or manic episodes (9%), and psychological or pharmacological issues. They also found that 60% of pilgrims sought psychiatric treatment before the Hajj pilgrimage, and 71% had never traveled, heightening isolation and anxiety. Alnabulsi and Drury (2014) verified pilgrims’ safety in relation to the concepts of social identity and crowding; 1,194 international pilgrims were screened at the Mina and Holy Mosque, with results demonstrating that pilgrims’ safety is affected by crowding, and that pilgrims who perceived the audience as brothers felt safer in large numbers, changing their social identity. That is, strong group identity minimized crowding anxiety, lowering risks amid the pilgrimage while enabling the exchange of identification in smaller groups. Khan et al. (2016) examined 136,000 Indian pilgrims with mental illnesses, finding that 182 pilgrims had moderate-to-severe mental difficulties and 22 (12%) were hospitalized, and that pilgrims who were worried engaged with the pilgrimage while receiving help from others. Additionally, they experienced stress and adaptation (45.7%), acute psychosis (9.8%), severe insomnia (7.3%), mood difficulties (5.6%), sleep problems (55%), fear and anxiety (45% and 41%, respectively), and crowd fright (27%). In another research, Hajj and its large crowds were new to most patients and rural residents, with 83% of patients (and 90% of women) being unaware of Hajj’s “difficulty” before arriving, causing concerns ( Alnabulsi et al., 2018). Alnabulsi et al. (2018) analyzed collaboration among 1,194 pilgrims by environment (Grand Mosque vs. courtyards), crowd density, and group identification, identifying that although the density outside was larger, pilgrims in the open arena collaborated and helped more than those inside the Grand Mosque. Therefore, community identification predicted collaboration more than crowd density, with the Grand Mosque setting seeming less representative of the social identity than the setting outside the common area.

Additionally, Almehmadi et al. (2021) looked into the health-risk perceptions and preventive health habits of 233 visitors coming from 28 nationalities, and they pointed out that only 24% of pilgrims used masks while 94% said the Hajj pilgrimage was safe, and 88% got health advice before going. This cited study shows some sort of mismatch between perceived safety and the real preventive behavior, so they recommended multilingual pre-travel health education in order to secure pilgrim safety too. Also, it seems that most studies dealing with crowd control during large-scale religious gatherings have leaned on engineering-oriented methods, logistical approaches, and infrastructure techniques rather than anything else ( N Alhawsawi et al., 2020).

Although they have produced fruitful outcomes, three key gaps remain, as follows: psychological features and risk perception have seldom been examined within a predictive framework, creating an epistemic vacuum; quantitative prediction research within the context of Arab and Muslim Hajj pilgrims is scarce; crowd-control techniques prioritize logistics over psychology. These shortcomings hinder the theoretical and practical knowledge within the context of crowd behavior research and high-density pilgrimage environments ( N Alhawsawi et al., 2020; Taibah et al., 2018).

The present study was designed to address these limitations. Based on social identity theory ( Tajfel & Turner, 1979) and Durkheim’s (2016) theory of collective effervescence, this study posits that pilgrims’ shared religious identity, spiritual engagement, and institutional trust function as psychosocial resources that mediate the translation of Risk perception into adaptive crowd behavior. The goals of the study were as described herein: (1) assess the levels of risk perception, psychosocial factors, and crowd behavior among Hajj pilgrims; (2) investigate the bivariate relationships among these variables; (3) evaluate the predictive contribution of risk perception and psychosocial factors to crowd behavior, while accounting for demographic characteristics.

## Methods

### Study design

A descriptive-analytical design with a predictive focus was deemed appropriate for the study’s goal of analyzing psychosocial aspects, risk perception, and pilgrims’ crowd behavior in holy places. This approach allowed for the study of the phenomenon as it occurred in reality and of the relationships between its variables without researcher intervention.

### Participants

To meet the study goals, all Muslim pilgrims who undertook the Hajj pilgrimage at Mina, Muzdalifah, Arafat, the Jamarat Bridge, and the Grand Mosque in Makkah throughout the study period were included. The group was varied in sex, age, education, country, and Hajj experience. Hajj is a pilgrimage event that attracts international pilgrims, such that its variation allows for studies on pilgrims’ psychology, sociology, risk perception, and crowd behavior. The pilgrim population’s sex, age, education, and culture were represented by 407 male and female pilgrims who partook in the research. The research required people with direct and meaningful Hajj or Umrah experience and who had gone to the holy sites; hence, it utilized purposive sampling. Only those who met the requirements were sampled to ensure data accuracy and relevance. Respondents were required to have performed Hajj or Umrah within five years (1442–1446 AH) to guarantee that their experiences were current and aligned with the holy site’s organizational and crowd management procedures. We assumed that both performing Hajj and Umrah participants were eligible. This is because the study was concerned with crowd exposure, perceived risks and psychosocial experiences related to movement and interaction in highly populated religious settings. While Hajj and Umrah are different in terms of magnitude and duration, they both provide experiences that are relevant to the study objectives on crowd behavior and risk perception. The five-year period was selected to ensure an adequate sample size while maintaining reasonable recall accuracy and consistency with recent Hajj organizational procedures.

The sample includes foreigners (i.e., Saudi, Arab, and non-Arab pilgrims) to ensure that the data reflects the Hajj and Umrah culture and demography, pilgrims’ mental, social, and communal traits, and secure the cultural and national variety characteristic of these international pilgrimages. In addition, the use of purposive online sampling may have introduced selection bias, as pilgrims who were more educated, digitally connected, or more willing to participate may have been overrepresented. Therefore, caution should be exercised when generalizing the findings beyond the characteristics of the respondents included in this study. Participation in the study was optional. The questionnaire link was distributed electronically through pilgrim networks, social media platforms, and personal contacts to reach individuals who had completed Hajj or Umrah during the specified period. Hajj and Umrah pilgrims from 1442–1446 AH took a Google Forms quiz instead of seeing the holy sites. This retroactive online technique was employed for logistical and safety reasons, with the downside being lower external validity, as replies may have been based on recalled experiences. The questionnaire’s participant information statement outlined the study’s goals, data confidentiality, and scientific use of the responses, and Digital written informed consent was obtained from all respondents prior to participation. Respondents were required to review the study information and provide their consent electronically before accessing the questionnaire., as voluntary participation was essential for the study’s goals.

### Measurements

To achieve the study’s objectives, the survey’s questionnaire included items on demographic variables (e.g., sex, age, educational level, and economic status) and three other instruments, which are outlined below.

***Psychological and Social Factors Scale***

A Psychological and Social Factors Scale based on Cheng et al. (2019) was used to assess the psychosocial factors of pilgrimage. The study team translated the English instrument into Arabic and had an independent bilingual expert back-translate to ensure conceptual comparability. The modified scale comprised 19 items on spirituality (6 items), learning (4 items), sharing (3 items), help (3 items), and dissatisfaction/negativity (3 items). Items were responded on a five-point Likert-type scale ranging from 1 (strongly disagree) to 5 (strongly agree), with higher scores implying an increased connection between pilgrims’ experiences and psychosocial characteristics.

*Validity and reliability*

In a confirmatory factor analysis, the five-dimensional, 19-item scale model fit the data well. The construct validity of the scale was confirmed through all fit indices being below the statistical thresholds. There was convergence between composite reliability and average variance extracted. The expert review established content validity for this research, with 80%–90% rater agreement. Regarding construct validity, Pearson’s correlation coefficients between the scores for the subdimensions and overall scale were significant at the 0.01 level. Cronbach’s alpha coefficients and split-half reliability scores were over 0.80 for all subdimensions and 0.93 for the total scale, demonstrating the scale’s good internal consistency. These data indicate that the scale was reliable and consistent, making it suitable for this study.

***Perceived Risk Scale Among Pilgrims***

To meet the study’s goals, we first reviewed the available scales in the literature on pilgrims’ risk perception ( Azmi et al., 2021; Cheng et al., 2019; Nautiyal et al., 2025; Sharifi-Tehrani & Esfandiar, 2018), disease risk perception, risk preventive effectiveness, the health belief model, and general risk perception scales. This led the researchers to develop the Perceived Risk Scale among Pilgrims. This is a comprehensive 23-item instrument created for assessing risk perception among Hajj pilgrims within the context of Arab culture and society, with items being divided into the six subdimensions of perceived probability, severity, anxiety, personal readiness, knowledge of risks, trust in institutions, and community support. Items were responded on a five-point Likert-type scale ranging from 1 (strongly disagree) to 5 (strongly agree), with higher scores implying a higher risk perception among pilgrims.

The items of the Perceived Risk Scale were developed following a comprehensive review of the literature on risk perception, pilgrimage-related risks, health-risk behavior, and preventive health models. Eight experts in psychology, measurement and evaluation and Hajj-related research reviewed an initial list of items to ensure that they were clear, relevant, culturally appropriate and covered the right topics. We revised some items and improved on them, based on their recommendations. The revised instrument was then pilot tested on a sample of 78 pilgrims with similar characteristics to that of the target study population. The pilot study enabled the improvement of item wording, clarity and comprehensibility prior to the administration of the final version in the main study.

*Validity and reliability*

Expert assessment demonstrated the scale’s content validity through an interrater agreement of over 80%, proving the usefulness of the questions for evaluating risk perception among Hajj pilgrims. Pearson’s correlation coefficients between the scores for the subdimensions and overall scale were significant at the 0.01 level, confirming its construct validity. Exploratory factor analysis found that the data were suitable (KMO = 0.91) and Bartlett’s test of sphericity was significant. Exploratory factor analysis supported the multidimensional structure of the scale and resulted in the retention of seven interpretable factors. The final factor structure was evaluated based on statistical adequacy, factor interpretability, and conceptual consistency with the theoretical dimensions underlying pilgrims’ risk perception. The seven retained factors collectively explained 68.4% of the total variance, with factor loadings ranging from 0.54 to 0.83.

Seven variables with factor loadings from 0.54 to 0.83 explained the variation clearly demonstrating their importance. Confirmatory factor analysis results revealed that all fit indices were within the acceptable limits. This confirmed the construct validity of the seven-factor model. High internal consistency suggests the scale is dependable, overall. For the subdimensions, Cronbach’s alpha values sat between 0.81 and 0.88, and for the entire scale, it came out to 0.93. Split half reliability was high as well, and the total scale coefficient was 0.91. Taken together, these results suggest that the scale is reliable, internally consistent and appropriate for use in this study.

***Hajj Crowd Behavior Scale***

Abd Rahman et al. (2019) developed the Hajj Crowd Behavior Scale, which measures pilgrims’ cognitive, emotional, and behavioral behaviors at holy sites and was subsequently translated and culturally adapted for the Arab environment. In Arabic, the scale has 52 items divided into the three subdimensions of cognitive (1–18), emotional (19–35), and behavioral (36–52). Items were responded on a five-point Likert-type scale ranging from 1 (strongly disagree) to 5 (strongly agree), with higher scores implying a higher occurrence of crowd behavior. Individual scores for each subdimension were added to obtain a total scale score, which indicated low, moderate, or high crowd behavior.

*Validity and reliability*

An examination by social psychology and crowd behavior experts confirmed the scale’s content validity, with an interrater agreement of 85%, showcasing that the items were culturally acceptable and reflected the three-crowd behavior subdimensions. Pearson’s correlation coefficients between the scores for the subdimensions and total scale indicated appropriate construct validity and coherence across the scale dimensions, varying from 0.72 to 0.81 (p < 0.01). Additionally, the scale had high internal consistency, with the Cronbach’s alpha for the cognitive, emotional, and behavioral subdimensions being 0.87, 0.89, and 0.84, respectively, and that of the total scale alpha being 0.91. The split-half reliability coefficients, ranging from 0.81 to 0.86 for subscales and 0.88 for the total score, confirmed the instrument’s robustness and stability. These results indicated that the Scale is a robust and reliable tool for assessing pilgrimage crowd behavior in field-based research.

***Overall Validity and Reliability of the Study Instruments***

Validity and reliability analysis showed that all the study tools had acceptable psychometric properties. The content validity was evaluated by expert assessment with agreement rates above the accepted threshold. Construct validity was supported by significant item-total and dimension-total correlations, exploratory and confirmatory factor analyses, and acceptable model fit indices. Reliability analysis showed that all the instruments had high internal consistency with Cronbach’s alpha and split-half coefficients above the recommended standards. Taken together, these findings support the use of the study instruments to measure psychosocial factors, risk perception and crowd behaviour among Hajj pilgrims.

Completed questionnaires were screened for completeness and consistency before analysis. Responses with substantial missing data or incomplete questionnaires were excluded. The final dataset was coded and entered into IBM SPSS Statistics (Version 28) for statistical analysis. Data were checked for accuracy, normality, and consistency prior to conducting the main analyses.

### Statistical analysis

Data management and statistical analyses were performed using IBM SPSS version 28 and AMOS version 22. The initial data screening process assessed data correctness, identified missing values and outliers, and verified distributional assumptions such as normality. Means, standard deviations, frequencies, and percentages were used to describe the sample and key research variables. Reliability and validity assessments were used to investigate the psychometric qualities of the instruments. Cronbach’s alpha and split-half reliability coefficients were used to assess the internal consistency of the scales. Construct validity was assessed using exploratory factor analysis with principal component extraction and varimax rotation. Confirmatory factor analysis was performed to test the model’s data fit, using standard goodness-of-fit indices, including χ ^2^/df, GFI, AGFI, CFI, TLI, RMSEA, and SRMR.

Pearson’s correlation coefficients were used to examine psychosocial characteristics, risk perception, and crowd behavior. The predictive ability of independent factors was assessed using multiple regression analysis, with crowd behavior as the dependent variable. All statistical tests were conducted at a significance level of p < 0.05 to ensure data interpretation accuracy and rigor.

## Results

### Demographic characteristics of respondents

Table 1 shows respondents’ demographic characteristics. Regarding nationality, 76.4% of sample members were non-Saudis, and the most representative age group was 30–39 years (44.5%), followed by 20–29 years (31.9%), and 40+ years (23.6%). Most of the sample had a bachelor’s degree (59.2%), followed by diplomas (24.3%) and postgraduate studies (16.5%). The highest proportion regarding economic level was medium (70.0%), followed by high (18.7%) and low (11.3%). These characteristics show that most of the sample was non-Saudi (76.4%), has 30–39 years old, holds a bachelor’s degree, and in the middle class. This multinational sample ensures that our study population accurately depicts the heterogeneity of the pilgrim community, supporting cross-national comparisons.

**Table 1.** Respondents’ demographic characteristics (N = 407).

Variable	Category	Repetition	Percentage (%)
Nationality	Non-Saudi	311	76.4
	Saudi	96	23.6
Age	20–29 years	130	31.9
	30–39 years	181	44.5
	40 +	96	23.6
Educational level	Diploma	99	24.3
	Bachelor	241	59.2
	Postgraduate studies	67	16.5
Economic level	Low	46	11.3
	Medium	285	70.0
	High	76	18.7

**Note**: Percentages are based on the total sample (N = 407).

### Levels of risk perception, psychosocial factors, and crowd behavior among pilgrims

**Figure 1.**
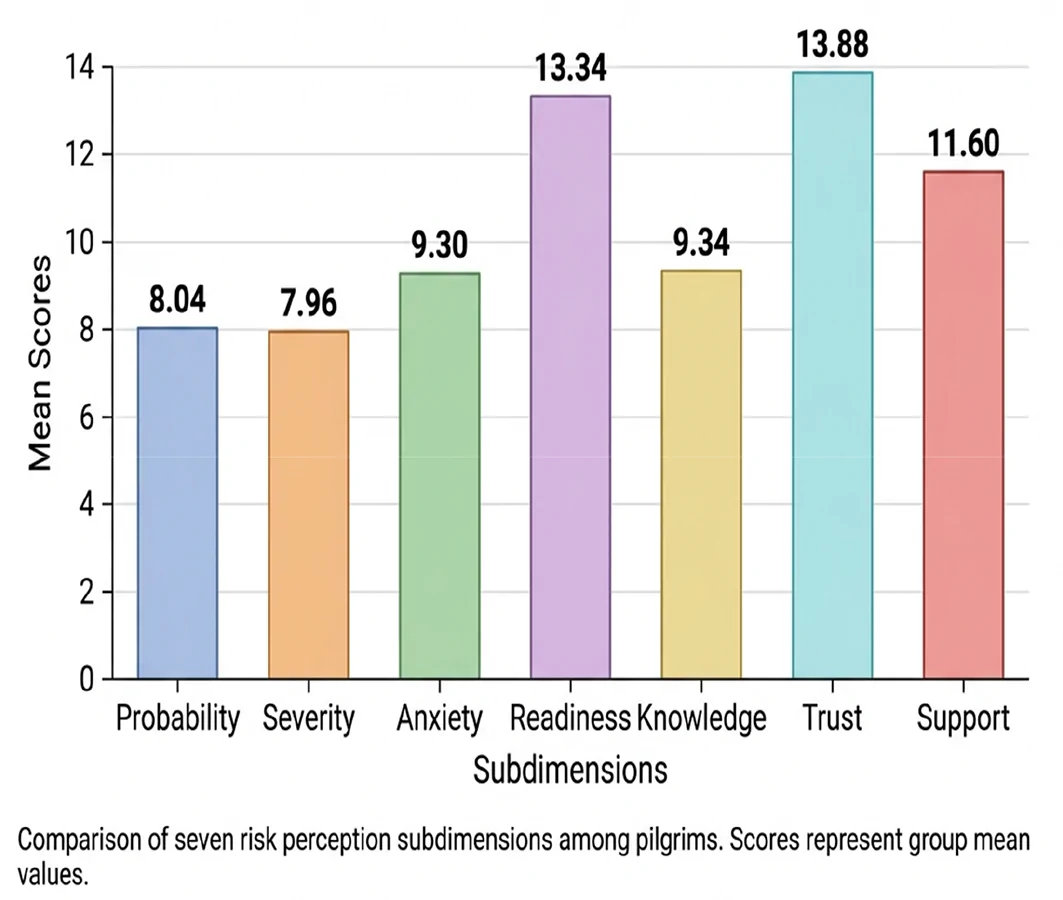
Mean scores of risk perception subdimensions among pilgrims. Error bars were not displayed because the figure was intended to present descriptive mean values only.

Figure 1 shows that pilgrims’ risk perception was moderate to high, with an average total scale score of 72.862 and a standard deviation (SD) of 18.286. The subdimension of trust in institutions ranked the highest, with an average of 13.877 (SD = 3.263), indicating pilgrims’ strong self-reported faith in Hajj organizers; the subdimension of personal readiness followed closely (M = 13.344, SD = 3.968), showing that the pilgrims understood the necessity of personal preparation and prevention. Pilgrim anxiety averaged at 9.310 (SD = 3.199). The perceived probability (M = 8.037, SD = 3.343) and severity (M = 7.956, SD = 3.424) subdimensions demonstrated balanced risk perception, while knowledge of risks averaged at 9.742 and community support at 10.597, highlighting the significance of knowledge awareness and solidarity in pilgrims’ safety.

For psychosocial factors, the total score was 64.408 (SD = 13.205), indicating an acceptable balance of scores for psychosocial factors. The spirituality subdimension showed the highest average scores (M = 20.383, SD = 4.876), indicating that the pilgrims drew positive psychological energy from their faith and spirituality. The learning subdimension followed with averages of 13.418 (SD = 2.890) and 11.091 (SD = 2.414), depicting that the pilgrims had prior training and experience in behavioral management amid pilgrimages and related rituals. The scores for help (M = 10.015, SD = 2.533) and dissatisfaction/negativity (M = 9.501, SD = 2.933) were lower, showing individual variances in cooperation and contentment, but within acceptable ranges. The total mean score for crowd behavior during pilgrimage was 213.025 (SD = 22.866).

### Crowd behavior subdimensions among pilgrims

**Figure 2.**
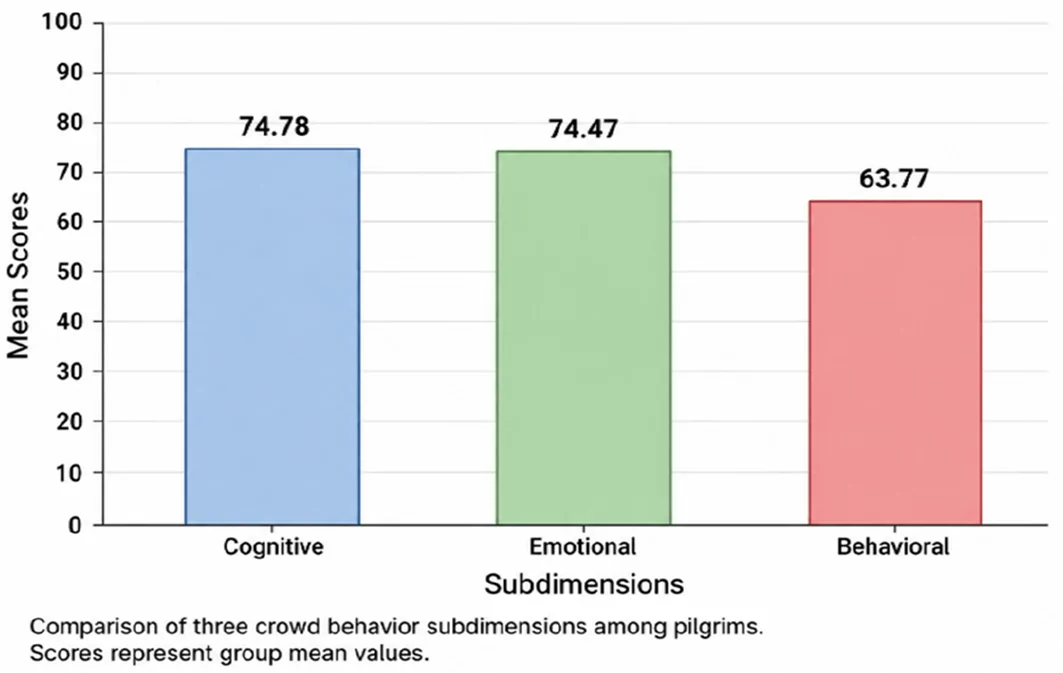
Mean scores of crowd behavior subdimensions among pilgrims.

Figure 2 shows that the cognitive subdimension had the highest mean score (M = 74.779, SD = 8.253), reflecting a high awareness and understanding of pilgrimages. The emotional subdimension followed closely (M = 74.472, SD = 9.304), indicating a strong emotional engagement during pilgrimage. The behavioral subdimension showed a considerably lower average score (M = 63.774, SD = 10.805), suggesting that the transfer of cognitive and emotional feelings into practical behavior may occasionally be influenced by crowds or environmental pressures.

These descriptive findings show that pilgrims have moderate-to-high Risk perception and institutional trust, a balanced psychosocial profile with spirituality as the dominant subdimension, and predominantly positive cognitive and emotional crowd behavior. The behavioral subdimension showed somewhat lower scores, likely because of the physical and environmental constraints of high-density pilgrimage settings.

### Psychosocial factors and risk perception among pilgrims

Table 2 shows that significant relationships (p < 0.01) exist between psychosocial characteristics and pilgrims’ risk perception. Spirituality was moderately correlated with all aspects, including perceived probability (r = 0.440), dissatisfaction/negativity (r = 0.555), and risk perception (r = 0.569), demonstrating a positive association between spirituality and risk perception.

**Table 2.** Relationship between psychological and social factors and risk perception among pilgrims during their performance at holy sites.

Variables/subdimensions	Perceived probability	Severity	Anxiety	Personal readiness	Knowledge of risks	Trust in institutions	Community support	Overall risk perception score
Spirituality	0.440 **	0.438 **	0.449 **	0.420 **	0.501 **	0.466 **	0.429 **	0.569 **
Learning	0.516 **	0.494 **	0.462 **	0.450 **	0.484 **	0.493 **	0.449 **	0.607 **
Sharing	0.259	0.332	0.295	0.348	0.377	0.473 **	0.411 **	0.450 **
Help	0.445 **	0.469 **	0.393 **	0.385 **	0.402 **	0.476 **	0.403 **	0.538 **
Dissatisfaction/negativity	0.555 **	0.571 **	0.550 **	0.465 **	0.431 **	0.405 **	0.270	0.595 **
Psychosocial factor total score	0.532 **	0.547 **	0.518 **	0.494 **	0.533 **	0.548 **	0.469 **	0.661 **

** p < 0.01 indicates statistical significance. Correlation coefficients are based on Pearson’s r.

The learning subdimension was one of the most influential, being associated with high values for most subdimensions, including perceived probability (r = 0.516) and risk perception (r = 0.607), demonstrating the importance of knowledge and learning in cognition. The sharing subdimension was weaker, revealing some non-significant correlations (e.g., r = 0.259 with perceived probability) and meaningful associations, such as with trust in institutions (r = 0.473) and total score (r = 0.450).

The help subdimension showed intermediate and significant associations with most subdimensions, including perceived probability (r = 0.445) and risk perception (r = 0.538), highlighting the importance of pilgrims’ mutual support in raising awareness. Dissatisfaction/negativity was evident and scored high for severity (r = 0.571) and total scores (r = 0.595), demonstrating that unpleasant experiences may increase risk perception. The scores for psychosocial factors had the highest correlation, ranging from the score for personal readiness (r = 0.494) to that of risk perception (r = 0.661), confirming that the total interaction of these subdimensions’ shapes pilgrims’ Risk perception more clearly and deeply.

### Associations between psychosocial factors and crowd behavior

The cognitive, emotional, and behavioral dimensions reported in Table 3 represent the three subdimensions of the Hajj Crowd Behavior Scale. Accordingly, the reported correlations reflect associations between psychosocial factors and different aspects of crowd behavior rather than dimensions of risk perception.

**Table 3.** Pearson’s correlation coefficients between psychosocial factors and the cognitive, emotional, and behavioral dimensions of crowd behavior.

Variables	Cognitive subdimension	Emotional subdimension	Behavioral dimension	Crowd behavior total score
Spirituality	0.287 **	0.298 **	0.400 **	0.414 **
Learning	0.271 **	0.311 **	0.370 **	0.399 **
Sharing	0.251 **	0.238 **	0.251 **	0.306 **
Help	0.292 **	0.304 **	0.359 **	0.399 **
Dissatisfaction/negativity	0.202 **	0.027	0.307 **	0.229 **
Total score of psychosocial factors	0.312 **	0.286 **	0.411 **	0.423 **

** p < 0.01 indicates statistical significance. Correlation coefficients are based on Pearson’s r.

As shown in Table 3, the spirituality subdimension was associated with the scores for behavioral dimension (r = 0.400) and total crowd behavior (r = 0.414), whereas the learning subdimension was associated with scores for cognitive behavior (r = 0.271) and total crowd behavior (r = 0.399). Psychosocial characteristics and the crowd behavior were marginally linked (r = 0.423). Regarding crowd behavior, the cognitive subdimension was strongly correlated with emotional (r = 0.740) and overall crowd behavior (r = 0.846). The emotional subdimension was substantially correlated with total score (r = 0.835), whereas the behavioral subdimension was correlated with value (r = 0.751). These findings confirm the internal coherence of the Hajj Crowd Behavior Scale and the coherent model of crowd behavior.

### The relative contribution of risk perception and psychosocial factors in predicting pilgrims’ crowd behavior

Based on multiple regression analysis, the suggested model with risk perception and psychosocial variables as predictors was statistically significant (F = 44.533, p < .001). The model explained 18.1% of the crowd behavior variation (R ^2^ = 0.181; adjusted R ^2^ = 0.177), indicating limited explanatory power. The model’s overall correlation coefficient (R = 0.425) and RMSE (20.749) demonstrated good prediction accuracy. The standardized regression coefficients showed that psychosocial variables significantly predicted crowd behavior (β = 0.391, p < .001). In the model including psychosocial variables, risk perception did not substantially predict crowd behavior (β = 0.049, p = .413). According to Table 4, psychosocial characteristics significantly predicted crowd behavior (β = 0.391, p < .001), but risk perception did not (β = 0.049, p = 0.413). This model suggests that psychosocial dynamics have a more significant effect than risk perception, although the low explained variance (R ^2^ = 0.181) suggests caution when generalizing beyond this group. Demographic variables may function as contextual markers reflecting cultural and resource differences rather than as direct behavioral determinants.

**Table 4.** Non-normative and standard regression coefficients for the predictive effects of risk perception and psychosocial factors on crowd behavior.

Variable	B non-standard	Standard error	Beta standard	t	p
(Intercept)	164.944	5.233	—	31.522	< .001
Risk perception	0.061	0.075	0.049	0.820	0.413
Psychosocial factors	0.677	0.104	0.391	6.517	< .001

**Note**: β = standardized coefficient; B = unstandardized coefficient; SE = standard error; p < 0.05 indicates statistical significance.

The low proportion of explained variance suggests that important influences on crowd behavior are not accounted for by the present model. Environmental conditions, crowd density, organizational arrangements, communication systems, previous experience of pilgrimage and situational characteristics may influence behavioural outcomes during Hajj. The future predictive models should integrate these factors for a better consideration of crowd behavior ( Fruin, 1993; Helbing et al., 2000; Still, 2014).

### Differences in risk perception, psychosocial factors, and crowd behavior by demographic variables

Table 5 presents the results of the between-subjects effects of demographic variables on risk perception, psychosocial factors, and crowd behavior. Nationality demonstrated the strongest effect across the three dependent variables. The effect size was moderate for psychosocial factors (η ^2^p = .087) and small-to-moderate for risk perception (η ^2^p = .050) and crowd behavior (η ^2^p = .048), indicating that nationality accounted for a meaningful proportion of variance in these outcomes. Economic level showed a statistically significant but small effect on psychosocial factors (η ^2^p = .016). In contrast, age and educational level exhibited negligible effect sizes and did not significantly contribute to differences in risk perception, psychosocial factors, or crowd behavior. These findings suggest that nationality was the most influential demographic characteristic in the present study, whereas the contribution of the remaining demographic variables was limited.

**Table 5.** Between-subjects effects of demographic variables on risk perception, psychosocial factors, and crowd behavior.

Source	Dependent variable	Total boxes (Type III SS)	df	Average squares (MS)	F	Sig	Partial η ^2^
**Nationality**	Risk perception	6656.042	1	6656.042	20.781	0.000	.050
	Psychosocial factors	5975.432	1	5975.432	37.844	0.000	.087
	Crowd behavior	9969.031	1	9969.031	19.970	0.000	.048
**Age**	Risk perception	544.979	2	272.490	0.851	0.428	.004
	Psychosocial factors	760.354	2	380.177	2.408	0.091	.012
	Crowd behavior	713.965	2	356.982	0.715	0.490	.004
**Educational level**	Risk perception	1.209	2	0.604	0.002	0.998	.000
	Psychosocial factors	448.525	2	224.262	1.420	0.243	.007
	Crowd behavior	761.674	2	380.837	0.763	0.467	.004
**Economic level**	Risk perception	284.750	2	142.375	0.445	0.641	.002
	Psychosocial factors	1015.254	2	507.627	3.215	0.041	.016
	Crowd behavior	2012.463	2	1006.231	2.016	0.135	.010
**Error**	Risk perception	127795.224	399	320.289	—	—	—
	Psychosocial factors	63000.633	399	157.896	—	—	—
	Crowd behavior	199179.890	399	499.198	—	—	—

**Note**: SS = sum of squares; df = degrees of freedom; MS = mean square; p < 0.05 indicates statistical significance.

*Partial eta squared (η ^2^p) was used as an effect size measure. Values of approximately .01, .06, and .14 indicate small, medium, and large effects, respectively.*

The one-way ANOVA results ( Table 5) demonstrated statistically significant differences by nationality for all the dependent variables (risk perception, psychosocial factors, and crowd behavior) with all values being significant at p < .001. However, post hoc comparisons were not necessary for this variable as it had only 2 groups. On the other hand, no significant differences were found in age and educational level for all dependent variables, indicating that these factors have little influence. Furthermore, only economic level was statistically significant (p = .041) on psychosocial factors, but no significant differences were observed for the rest of the variables. Thus, post hoc comparisons (Tukey HSD test) were performed to determine the direction of the differences ( Table 6). The results showed differences between the low and the medium economic groups, with the medium economic groups having better results.

**Table 6.** Post Hoc (Tukey HSD) comparisons of psychosocial factors by economic level.

Economic level	Mean (M)	SD	Comparison	Mean Difference (I−J)	Std. Error	*p*	95% CI
Low	61.20	12.50	Low vs Medium	**-5.30**	2.05	**.041** *	-10.50, -0.10
Medium	66.50	13.00	Low vs High	-3.90	2.10	.089	-9.20, 1.40
High	65.10	12.80	Medium vs High	1.40	2.00	.640	-3.60, 6.40

* p < .05.

Post hoc comparisons were conducted using Tukey HSD test following a significant ANOVA result for psychosocial factors across economic levels. Significant differences were found only between the low and medium groups, in favor of the medium group.

## Discussion

### The first main finding: pilgrims’ psychosocial factors and risk perception

Pilgrims evaluated the Hajj pilgrimage’s risks as moderate to high in this study. Risk perception was dominated by an influence of trust in institutions and personal readiness, whereas anxiety, perceived probability, and severity matched. Pilgrims tend to be more mentally balanced and spiritual, such that the skillfully organized and spiritually important Hajj may present risks regarded as manageable rather than scary. This finding complements those of previous studies, which found that institutional trust, shared religious identity, and spirituality affect risk evaluations during large religious gatherings. Alnabulsi and Drury (2014) discovered that pilgrims who trusted organizational authorities and strongly identified with the crowd felt safer in large crowds. According to collective pilgrimage studies, a shared spiritual identity and emotional synchrony decrease anxiety and enhance adaptive responses to perceived risk by increasing psychological comfort, social cohesiveness, and emotional stability. Samarkandi et al. (2025) also showed that institutional trust and prior pilgrimage experience moderate pilgrims’ risk perceptions, resulting in greater readiness and compliance rather than fear-based reactions. These findings differ from those of research that found pilgrims underestimating health risks or weakly translating Risk perception into preventive behaviors. Almehmadi et al. (2021) observed that pilgrims were aware of health hazards, but rarely took precautions, while Khan et al. (2016) discovered that first-time pilgrims, particularly those unaccustomed to large crowds, reported increased tension, anxiety, and adjustment concerns. These differences in evidence may owe to divergences in organizational readiness, health communication tactics, pilgrims’ experience levels, and data collection scheduling (relative to important global health crises) among these studies.

These results can be analyzed through the lenses of social identity theory and contemporary crowd psychology. When people see themselves as part of a religious group and trust the legitimate organizational structures, risk perception is controlled by society rather than improved by individuals ( Alnabulsi & Drury, 2014). Durkheim’s collective effervescence theory posits that shared rituals and emotional synchronization offer reassurance, moral regulation, and social order within collective settings. Indeed, spirituality enables individuals to reinterpret peril as a significant and controllable aspect of communal religious experiences, enhancing psychological resilience and fostering balanced crowd behavior in high-density pilgrimage settings ( Alnabulsi & Drury, 2014; Páez et al., 2015).

### The second main finding: pilgrims’ crowd behaviors

The study indicated that pilgrims exhibited stronger cognitive and emotional crowd behavior, but lower behavioral activity. Although pilgrims appear alert, understanding, and emotionally immersed in rites, other aspects of pilgrimages such as congestion, limited mobility, and weariness may also affect them. These findings confirm those of earlier studies indicating that shared religious knowledge and emotional synchrony boost pilgrims’ communal awareness and emotional coherence, especially in crowds ( Alnabulsi & Drury, 2014; Wlodarczyk et al., 2020). As described in religious crowd studies, cognitive and emotional convergence precedes behavioral coordination, particularly when the circumstances limit agency; emotional engagement and behavioral cooperation may be higher in less crowded or regulated settings ( Alnabulsi et al., 2018). This is explained by crowd psychology theories that divide cognition, emotion, and behaviors. Collaboration is based on shared identity and emotions, while external factors influence behavior, entailing those situational restrictions—not desire or awareness—can explain the gap observed in the current study between cognitive–emotional participation and behavioral expression, making environmental and crowd control essential to ensure adaptive crowd behaviors.

### The third main finding: pilgrims’ psychosocial factors, risk perception, and crowd behaviors

This study discovered substantial positive relationships among psychosocial characteristics, risk perception, and crowd behavior. Spirituality, learning, help, and psychosocial balance affected the crowd cognitive, emotional, and behavioral adaptations. Therefore, specific psychosocial dynamics were positively associated with cognitive, emotional, and behavioral crowd adaptation during religious pilgrimages like the Hajj. These descriptions complement those of prior studies, where a balanced risk perception in a supportive psychological and spirituality context improves adaptive group behavior over fearful or panicky behavior ( Alnabulsi et al., 2018). In high-density religious meetings, shared identification and emotional synchronization foster collective attentiveness and social norms ( Wlodarczyk et al., 2020). Health-related risk perception research shows that awareness, trust, and social support are more likely to affect cooperative and preventive behavior than Risk perception alone ( Almehmadi et al., 2021). This research supports social identity–based crowd behavior theories that imply shared identity, emotional cohesiveness, and authoritative legitimacy motivate collective action. These findings support the idea that psychological resources modify or mediate risk perception and adaptive behavior is supported, albeit formal mediation analyses are needed in the future to corroborate this influence pathway ( Alnabulsi et al., 2018; Wlodarczyk et al., 2020).

### The fourth main finding: pilgrims’ psychosocial factors and risk perception prediction

Psychosocial factors predicted crowd behavior better in the regression analysis. As a bivariate factor, risk perception was strongly correlated with crowd behavior, whereas psychosocial factors minimized its independent contribution, indicating mediation or suppression. The model explained 18.1% of the crowd behavior variance, with a small impact size (R ^2^ = 0.181). This difference may owe to factors such as Hajj experience, group dynamics, environment, and situational demands. These data suggest substantial psychosocial pathways, not all collective behavior features. Research has shown that Risk perception alone was not independently associated with adaptive crowd behavior in the present model, learning, and social support. Researchers show that psychosocial resources enhance community awareness via risk knowledge, not terror ( Reicher et al., 1995; Wlodarczyk et al., 2020), while health and disaster psychology research suggests that risk perception drives action indirectly through mediating variables such as institutional trust, perceived efficacy, and social norms ( Wlodarczyk et al., 2020). Additionally, psychosocial factors may affect risk perception in highly structured religious pilgrimages such as the Hajj, where institutional presence and community connections matter. According to social identity-based and psychosocial crowd behavior theories, community meaning, emotional congruence, and normative expectations influence crowd behavior more than risk assessment, while institutional trust, learning, and solidarity affect adaptive crowd behavior in pilgrimages. These discussions underline how crowd control success requires risk communication and psychological interventions. Since the model in this study explained 18.1% of the variance, future research should uncover additional factors that can influence crowd behaviors ( Alafif et al., 2025; Knoke, 2025; Shah, 2025).

### The fifth main finding: pilgrims’ demographic factors influencing risk perception, psychosocial factors, and crowd behavior differences

Nationality was the biggest demographic factor affecting risk perception, psychosocial factors, and crowd behavior. Age and education did not significantly affect any dependent variable, whereas economic level only affected some the psychosocial elements. These results find consonance in prior literature, indicating that age and education serve as background characteristics rather than direct predictors of crowd behavior in highly-regulated religious contexts ( Reicher, 2011). Standardized rituals, behavioral norms, and robust institutional governance may mitigate age- and educational-related behavioral disparities in large religious pilgrimages and assemblies, such as the Hajj, leading to more uniform behavior. Studies have indicated that nationality, culture, language, and previous exposure to significant religious events influence risk perception and psychological experiences ( Abd Rahman et al., 2019; Khan et al., 2016), as pilgrims from different nations may have distinct crowd management systems, authority trust, and risk interpretations that may affect their psychological responses and crowd behavior.

Based on the current findings, economic limitations may influence people’s psychosocial factors and cause resource, housing, and comfort inequities that indirectly harm Hajj pilgrims’ emotional well-being and social experiences. Still, religious affiliation and institutional governance may affect crowd behavior more than economic level. These results support demographic factors as contextual indicators, not causal ones. In highly-salient shared identity settings, such as the Hajj pilgrimage, social identity theory states that group-based norms and meanings matter more than demographics ( Norenzayan & Shariff, 2008; Petrica et al., 2024). Additionally, effective crowd management should prioritize culturally sensitive communication and psychological interventions that account for national and cultural diversity above age- or education-based distinctions ( Norenzayan & Shariff, 2008; Reicher, 2011). Many risk psychology and crowd behavior studies also emphasize psychosocial factors over risk perception, corroborating these descriptions findings. Studies on risk psychology and crowd behavior have shown that trust, shared identity, meaning-making, and collective effectiveness influence risk perception and action ( Wlodarczyk et al., 2020; Alnabulsi et al., 2018). Wlodarczyk et al. (2020) specifically proposed a dynamic risk perception model in which identity processes, perceived control, and the social environment influence behavior more than perceived probability or severity. Alnabulsi and Drury (2014) also demonstrated that social norms, institutional trust, and collective meaning are more reliably associated with adaptive conduct than risk perception alone. Psychosocial resources may help explain the association between risk perception and coordinated group behavior during religious pilgrimages such as Hajj, fostering cooperation rather than fear or avoidance during religious pilgrimages like Hajj.

Crowd science and behavior research support these findings. Drury and Reicher (2000) argued that, in dense crowds, collective organization and social regulation govern behavior more than individual cognition. That is, in structured situations such as the Hajj pilgrimage, similar norms, ritualized mobility, and institutional supervision decrease the behavioral relevance of risk assessment discrepancies. This proposal by Drury and Reicher’s (2000) is therefore consistent with the current findings that psychosocial factors are more proximally associated with crowd behavior than risk perception. Importantly. research on religion and resiliency also supports this study’s emphasis on spirituality. Regarding risk communication, Raj (2015) recommended prioritizing trust, cultural resonance, and shared meaning over technical risk statistics, while Taibah et al. (2018)—in their investigation of Hajj pilgrims’ communication choices—described that culturally resonant and spiritually themed communications outperformed technical danger information. This implies that trust building, culturally relevant messaging, and spirituality framing may improve adaptive crowd behavior more than quantitative risk knowledge.

## Conclusions

This study reports that risk perception and psychosocial variables can affect the crowd behavior of Hajj pilgrims, with factors like trust in institutions, learning experiences, and spiritually based reassurance strongly showing effects ( Alnabulsi & Drury, 2014; Páez et al., 2015). As risk perception was associated with crowd behavior but did not significantly predict it when psychosocial factors were included, this study proposes that crowd management strategies may benefit from incorporating psychological, social, and spiritual factors into their design and placing them side-by-side with physical and organizational approaches. In delivering these pieces of evidence, this work provides a context-specific, exploratory, predictive framework grounded in empirical data for studying crowd behavior at large religious gatherings, contributing to the literature on crowd behavior and risk perception. Risk communication tactics that promote trust in institutions, personal readiness, and include culturally and religiously relevant messaging may increase pilgrims’ compliance with preventive measures and safer crowd behaviors ( Slovic, 1987; Almehmadi et al., 2021). Regulators and mass event organizers stand to benefit from the guidance presented here, and the discussions deliver a useful framework for predictive and intervention-based crowd safety and well-being models.

### Limitations and strengths

Despite various contributions, this study has methodological limitations. First, the cross-sectional methodology prevented causal inferences between risk perception, psychosocial factors, and crowd behavior. Second, social desirability or response bias may affect self-reported measurements, all study variables were measured using self-report questionnaires administered at a single point in time. Therefore, the possibility of common method bias cannot be completely excluded. This may have contributed to inflated associations among some study variables, and the findings should be interpreted with this methodological consideration in mind. particularly in religious and highly controlled contexts, such as the Hajj pilgrimage. Third, the sample varied in country and demography, but it may not completely represent pilgrims’ temporal changes across Hajj seasons or organizational settings.

In addition, the use of purposive online sampling may have introduced selection bias, as pilgrims who were more educated, digitally connected, or more willing to participate may have been overrepresented. Furthermore, no formal comparison was conducted between the study sample and the broader global pilgrim population. Consequently, prudence is advised when extrapolating the findings beyond the attributes of the respondents in this study.

Also, nationality was analyzed as a broad Saudi versus non-Saudi category. Although this approach facilitated statistical comparison, it may have obscured important cultural, linguistic, and regional differences among international pilgrims. Future research may benefit from examining more detailed nationality or cultural group classifications to better understand the diversity of pilgrims’ experiences, perceptions, and crowd behaviors.

This study’s strengths include being among the few empirical investigations that combine risk perception and psychological factors to predict crowd behaviors during the Hajj pilgrimage. Valid and culturally tailored assessment tools, along with rigorous multivariate statistical analyses, increase the dependability and believability of the results, showing that incorporating psychological, social, and spiritual aspects into crowd management interventions can lead to more comprehensive strategies. With these qualities, this study advances crowd behavior theory and practice in large religious events. Furthermore, because respondents reported their experiences retrospectively, recall bias cannot be completely excluded.

No formal comparison was conducted between the study sample and the broader global pilgrim population. Therefore, the findings should be interpreted with caution when generalized beyond the characteristics of the respondents included in this study.

### Recommendations

Among risk perception factors, trust in institutions showed the highest scores. Therefore, crowd management authorities should make efforts to deliver clear, multilingual safety communication ( Almehmadi et al., 2021). Digital signs at pilgrimage locations and official cell phone alerts may help spread safety information quickly. Personal readiness ranked second among the risk perception subdimensions, entailing that brief pre-Hajj psychoeducational interventions, such as video-based instructional materials or structured workshops on crowd safety, first aid, and personal protective equipment, may improve readiness and reduce stress. Spirituality was the highest-scoring psychosocial subdimension; therefore, risk communication strategies that ground safety behaviors in the Islamic principle of self-preservation may increase pilgrims’ compliance with preventive measures. Finally, because learning is an important psychological feature, scalable digital technologies that supply pre-travel risk education in different languages may help the heterogeneous international Hajj community behave more informedly and adaptively.

## Ethical considerations

This study was approved by the Graduate Studies and Scientific Research Committee of the College of Social Sciences, Department of Psychology, Imam Mohammad Ibn Saud Islamic University (IMSIU), Riyadh, Saudi Arabia. Approval Number: [1193]. All procedures performed in this study involving human respondents were conducted in accordance with the ethical standards of the institutional research committee and with the principles of the Declaration of Helsinki. Respondents were informed about the purpose of the study, their right to withdraw at any time, and the confidentiality of their responses prior to participation.

## Data Availability

Data supporting the findings of this study are not publicly available due to ethical restrictions of privacy and confidentiality of human respondents. The study was approved by the Graduate Studies and Scientific Research Committee at Imam Mohammad Ibn Saud Islamic University, which permits the controlled sharing of data to maintain the confidentiality of the participants. De-identified data may be made available upon reasonable request for research verification or replication for qualified researchers, subject to ethical approval and institutional requirements. Requests to access the datasets should be directed to the corresponding author at ymkatatbh@imamu.edu.sa and include a brief description of the intended research purpose.
