# Supplementary material for: Prediction of Crowd Behavior in Holy Sites: Psychological, Social, and Risk Perception Factors among Pilgrims 
Source: F1000Res. 2026 Jul 22;15:828. Originally published 2026 May 28. [Version 2] doi: 10.12688/f1000research.180247.2 (PMC13560563; doi:10.12688/f1000research.180247.2)
Supplement: Peer review history

# Reviewer report: Reviewer response for version 2

**DOI:** 10.5256/f1000research.204949.r504855

**Published:** 2026-09-22

**Authors:** Sara Vestergren

**Affiliations:** 1 University of Reading, Reading, England, UK

As requested, this review is taken into account the revisions in relation to previous reviews, as well as providing a new review of the current manuscript. I appreciate the authors’ revisions and the importance of understanding pilgrims’ experiences and behaviour in large religious gatherings. The added information about recruitment, questionnaires, statistical results, and limitations is helpful. However, important concerns remain about what the questionnaires measure, how crowd psychology is used, the accuracy of the references, and some inconsistencies in the results. I therefore recommend further major revision.

 The manuscript describes a 52-item questionnaire covering thoughts, emotions, and behaviour. Higher scores are described as showing more crowd behaviour (p. 6). As I understand it, elsewhere these scores are taken to mean more adaptive behaviour, cooperation, and safety and these are different things. I recommend providing the full questionnaire, examples from each section, and clear scoring instructions, including any items scored in reverse. The authors should explain why a higher score means more adaptive behaviour. Does the questionnaire distinguish helping others, following instructions, feeling distressed, and behaving dangerously? Strong emotions or frequent thoughts about the crowd do not necessarily mean safer behaviour.

 The source of the questionnaire also needs checking. Abd Rahman et al. (2019), which the authors cite, describes a focus group with 11 Pakistani women. However, another paper from 2017 describes the development of a 52-item Hajj Crowd Behavior scale and states that further testing was needed. Please identify the exact source for the questionnaire used, correct the reference, and explain how it was adapted into Arabic.

 The risk-perception measure combines perceived probability, severity, and anxiety with preparedness, knowledge, institutional trust, and community support. These dimensions may relate to risk in different ways. For example, someone could believe a situation is dangerous while also feeling prepared and trusting the organizers. Please explain why these dimensions can be combined into a single score and what a higher total means. There may also be overlap between community support in this questionnaire and sharing and helping in the psychosocial questionnaire. The full items would help readers assess this.

 The source of the psychosocial questionnaire also needs clarification. Cheng et al. (2019) examines tourism-related constructs, and it is unclear how the five dimensions used here were derived from that study. Please explain the adaptation and how dissatisfaction/negativity was scored within the total. 

 The discussion draws on shared social identity to explain the findings. However, the measures described include spirituality, and it is unclear whether identification with other crowd members was assessed separately. Please clarify which questions, if any, measured shared identity. If it was not directly measured, its role should be presented as a possible explanation rather than a finding established by this study.

 The distinction between feeling safe and being physically safe also needs attention. On p. 4, the account of Alnabulsi and Drury (2014) moves from reduced crowding anxiety to 'lowering risks'. That study examined reported feelings of safety it did not demonstrate reduced injury risk.

 There are specific discrepancies that needs attention in relation to the developing argument:

- On p. 4, the manuscript states that Alnabulsi et al. (2018) found greater density outside the Grand Mosque. The original study reports higher estimated density inside: 5.63 people/m² compared with 4.96 outside. 
- On p. 14, Wlodarczyk et al. (2020) is credited with a risk-perception model comparing psychosocial processes with perceived probability and severity. The cited paper develops and tests a measure of emotional synchrony; it does not establish this comparison. 
- On pp. 14–15, the manuscript cites Taibah et al. (2018) as showing that spiritually themed communications outperformed technical danger information. However, that study examined pilgrims’ preferences for communication channels and their perceived effectiveness. It did not compare spiritual and technical message content or their effects on behaviour. Please explain, revise or provide a source that directly supports it.

The authors acknowledge the cross-sectional design and the model’s limited explanatory power. However, p. 11 still claims 'good prediction accuracy', although no validation on separate data is reported. Page 14 also describes the regression pattern as 'indicating mediation or suppression', despite acknowledging on p. 13 that mediation analysis is still needed. Please clarify or revise.

Is the work clearly and accurately presented and does it cite the current literature?

No

If applicable, is the statistical analysis and its interpretation appropriate?

Partly

Are all the source data underlying the results available to ensure full reproducibility?

Partly

Is the study design appropriate and is the work technically sound?

Yes

Are the conclusions drawn adequately supported by the results?

Partly

Are sufficient details of methods and analysis provided to allow replication by others?

No

Reviewer Expertise:

Social psychology, crowd psychology, collective behaviour, collective action

I confirm that I have read this submission and believe that I have an appropriate level of expertise to state that I do not consider it to be of an acceptable scientific standard, for reasons outlined above.

# Reviewer report: Reviewer response for version 2

**DOI:** 10.5256/f1000research.204949.r503725

**Published:** 2026-08-04

**Authors:** Mariusz Pecio

**Affiliations:** 1 Fire University, Słowackiego, Warsaw, Poland

The authors have addressed the majority of my previous comments and the manuscript has been substantially improved. In particular, the theoretical background has been strengthened by incorporating key literature from crowd science, the distinction between risk perception and risk awareness has been clarified, and the methodology has been described in much greater detail. The reporting of psychometric validation and statistical analyses has also been improved, and the interpretation of the regression results is now more appropriately aligned with the cross-sectional nature of the study.

 A few limitations remain. The predictive model explains a relatively small proportion of the variance in crowd behavior, indicating that important physical and environmental determinants of crowd dynamics are not captured by the current model. Although the authors now acknowledge this limitation, it should continue to be emphasized when interpreting the findings. In addition, several sections of the manuscript could benefit from minor language editing to improve readability and ensure a consistently formal scientific style.

 Overall, I appreciate the authors’ careful and constructive response to the review comments. The revised manuscript represents a clear improvement over the original version, and I have no major remaining concerns.

Is the work clearly and accurately presented and does it cite the current literature?

Partly

If applicable, is the statistical analysis and its interpretation appropriate?

Partly

Are all the source data underlying the results available to ensure full reproducibility?

No

Is the study design appropriate and is the work technically sound?

Partly

Are the conclusions drawn adequately supported by the results?

Partly

Are sufficient details of methods and analysis provided to allow replication by others?

Partly

Reviewer Expertise:

My research focuses on fire safety engineering, with particular emphasis on human evacuation, crowd dynamics during emergencies, and performance-based fire safety solutions.

I confirm that I have read this submission and believe that I have an appropriate level of expertise to confirm that it is of an acceptable scientific standard.

# Reviewer report: Reviewer response for version 1

**DOI:** 10.5256/f1000research.198842.r489625

**Published:** 2026-06-24

**Authors:** Mariusz Pecio

**Affiliations:** 1 Fire University, Słowackiego, Warsaw, Poland

Is the work clearly and accurately presented, and does it cite the current literature?

**Partly**

 The manuscript is generally well written and clearly structured. However, the theoretical framework requires further development, particularly through the inclusion of foundational crowd science literature and a clearer distinction between risk perception and risk awareness.

 Is the study design appropriate and is the work technically sound?

**Partly**

 The cross-sectional correlational design is appropriate for the study objectives. Nevertheless, the use of purposive sampling and retrospective self-reported experiences limits the generalizability and robustness of the findings.

 Are sufficient details of methods and analysis provided to allow replication by others?

**Partly**

 The methodology is described in reasonable detail, but additional information on instrument development, factor analyses, participant recruitment, and data handling procedures would improve reproducibility.

 If applicable, is the statistical analysis and its interpretation appropriate?

**Partly**

 The statistical methods are generally suitable. However, reporting of effect sizes, post hoc comparisons, and multicollinearity diagnostics is needed, and the predictive power of the regression model should be interpreted more cautiously.

 Are all the source data underlying the results available to ensure full reproducibility?

**No**

 The dataset is not publicly available and can only be obtained upon request from the corresponding author, which limits full reproducibility.

 Are the conclusions drawn adequately supported by the results?

**Partly**

 The conclusions are broadly consistent with the results, but some interpretations imply causal relationships that cannot be supported by the cross-sectional design.

 Overall Recommendation

**Major Revisions**

 This manuscript addresses an important and practically relevant topic and contributes to the understanding of crowd behavior during large religious gatherings. However, several issues should be addressed before indexing. The authors should strengthen the theoretical framework, clarify key concepts such as risk perception and risk awareness, provide more detailed methodological and psychometric information, improve statistical reporting, increase transparency regarding data availability, and moderate causal interpretations of the findings. Addressing these issues would substantially improve the scientific rigor and overall contribution of the study.

Is the work clearly and accurately presented and does it cite the current literature?

Partly

If applicable, is the statistical analysis and its interpretation appropriate?

Partly

Are all the source data underlying the results available to ensure full reproducibility?

No

Is the study design appropriate and is the work technically sound?

Partly

Are the conclusions drawn adequately supported by the results?

Partly

Are sufficient details of methods and analysis provided to allow replication by others?

Partly

Reviewer Expertise:

My research focuses on fire safety engineering, with particular emphasis on human evacuation, crowd dynamics during emergencies, and performance-based fire safety solutions.

I confirm that I have read this submission and believe that I have an appropriate level of expertise to confirm that it is of an acceptable scientific standard, however I have significant reservations, as outlined above.

# Reviewer report: Reviewer response for version 1

**DOI:** 10.5256/f1000research.198842.r491053

**Published:** 2026-06-18

**Authors:** Lisa Charmaine Welthagen

**Affiliations:** 1 Tourism Management, Tshwane University of Technology, Pretoria, Gauteng, South Africa

The topic of study is highly relevant, particularly given the increasing importance of understanding pilgrims' risk perception, psychosocial experiences, and crowd behaviour in large-scale religious gatherings. However, the literature review requires further development to provide a stronger theoretical foundation for the study. Several important theoretical perspectives appear to have been omitted, limiting the study's ability to justify the proposed relationships and provide a more robust basis for discussing and interpreting the results.  For example, a stronger literature introduction should demonstrate a clear progression from crowd science and psychological theory to risk perception and religious context, culminating in a justified need for an integrated predictive model of crowd behaviour in holy sites.  The introduction should first provide background on religious pilgrimage events, followed by a discussion of crowd dynamics and behaviour in mass gathering contexts. Key foundational contributions to crowd dynamics, including the work of Still, Fruin, and Helbing, should be integrated into the literature to provide a good theoretical foundation for understanding crowd behaviour within the study context. From here, the psychological factors/predictors should be discussed once again, supported by theories (Tajfel & Turner; Drury). Risk perception in mass gatherings should also be discussed in terms of perceived risk. There is confusion later in the discussion of the findings regarding risk perception (Slovic) and risk awareness. Although related, these are two different concepts.

 A clear aim of the study should be indicated to highlight the gap in research with distinct alignment of the following: From the article, it seems the following:

 Descriptive: Profile pilgrims and their perceptions

 Correlational: Relationship between risk perception and behaviour

 Predictive: Whether psychological/social factors predict crowd behaviour

 METHODOLOGY

 The following suggestions are made under the methodology section:

- Demographics Table should not be under participants, but instead under the findings section, as this reflects the findings and interpretation.
- Many of the discussions mentioned in the findings section are not discussed or supported in the literature.
- The reliability and validity should have their own section of discussion for the overall study
- Respondents are for quantitative and participants for qualitative
- Table 3 raises concerns and requires clarity- **What exactly do the cognitive, emotional, and behavioural dimensions measure?** If they are dimensions of *crowd behaviour*, then the discussion should be framed in terms of relationships with crowd behaviour. If they are dimensions of *risk perception* *, *the interpretation would be different.
- Table 4 heading refers to risk perception, but risk awareness is mentioned as a variable.
- agreement of reviewer 1 feedback regarding statistical analysis

Is the work clearly and accurately presented and does it cite the current literature?

No

If applicable, is the statistical analysis and its interpretation appropriate?

Partly

Are all the source data underlying the results available to ensure full reproducibility?

Partly

Is the study design appropriate and is the work technically sound?

Yes

Are the conclusions drawn adequately supported by the results?

Partly

Are sufficient details of methods and analysis provided to allow replication by others?

Partly

Reviewer Expertise:

event management, event risk management, wellness, tourism

I confirm that I have read this submission and believe that I have an appropriate level of expertise to confirm that it is of an acceptable scientific standard, however I have significant reservations, as outlined above.

# Reviewer report: Reviewer response for version 1

**DOI:** 10.5256/f1000research.198842.r489622

**Published:** 2026-06-13

**Authors:** Osama A Samarkandi

**Affiliations:** 1 King Saud University, Riyadh, Riyadh Province, Saudi Arabia

Reviewer Report

 This manuscript addresses an important and underexplored topic concerning crowd behavior during Hajj and examines the influence of psychosocial factors and risk perception on adaptive crowd behavior among pilgrims. The study is relevant to mass gathering medicine, crowd management, behavioral science, and pilgrimage safety. The manuscript is generally well organized, the sample size is adequate, and the authors attempt to integrate psychological and social theories into a predictive framework.

 The study has several strengths. It addresses an important operational issue in one of the world's largest recurring mass gatherings, includes a multinational sample of pilgrims, and evaluates psychosocial dimensions that are often neglected in engineering-based crowd management studies. The authors also provide psychometric information regarding the instruments used and attempt to move beyond descriptive analyses by incorporating regression modeling.

 However, several issues should be addressed before the manuscript can be fully endorsed.

 Major Comments

1. Clarification of the sampling strategy and study population

The manuscript states that purposive sampling was used and that participants completed an online retrospective questionnaire regarding Hajj or Umrah experiences within the previous five years. This approach introduces potential recall bias and selection bias. Participants who are more educated, digitally connected, or psychologically engaged may have been more likely to respond. The authors should discuss these limitations in greater detail and clarify how participants were recruited and whether any attempt was made to compare respondents with the broader pilgrim population.

1. Limited predictive power of the regression model

The primary regression model explained only 18.1% of the variance in crowd behavior (R² = 0.181). Although statistically significant, this indicates that most determinants of crowd behavior remain unexplained. The discussion should more clearly acknowledge the modest explanatory power of the model and avoid overstating predictive capability. Additional contextual, environmental, organizational, and situational variables are likely important contributors to crowd behavior.

1. Interpretation of causality

The manuscript repeatedly uses language suggesting influence or prediction. Because the study is cross-sectional and observational, causal interpretations should be avoided. The findings demonstrate associations rather than causal effects. Statements indicating that psychosocial factors "influence" crowd behavior should be softened and framed as associations.

1. Development of the Perceived Risk Scale

The Perceived Risk Scale among Pilgrims appears to have been developed specifically for this study. While reliability and validity statistics are reported, additional information is needed regarding item development, expert review procedures, pilot testing, factor retention criteria, and the complete factor structure. The manuscript would be strengthened by providing the full instrument as supplementary material.

1. Nationality effects require deeper exploration

Nationality emerged as the strongest demographic variable across several analyses. However, nationality is treated largely as a binary Saudi versus non-Saudi variable. Given the cultural heterogeneity of Hajj pilgrims, this classification may mask substantial variation between national groups. The authors should discuss this limitation and consider whether broader cultural, linguistic, or regional categories may provide more meaningful interpretation.

1. Common method bias

All variables were collected using self-report questionnaires administered at a single point in time. This raises the possibility of common method variance inflating observed correlations. The authors should acknowledge this limitation and discuss its potential impact on the findings.

 Minor Comments

1. The manuscript would benefit from language editing to improve clarity and readability.
2. Several sections of the Discussion contain repetitive explanations of social identity theory and collective behavior concepts and could be condensed.
3. The distinction between Hajj and Umrah participants should be clarified, as both groups appear eligible for inclusion.
4. The rationale for selecting the five-year recall period (1442–1446 AH) should be explained.
5. The manuscript would benefit from reporting effect sizes alongside significance tests for group comparisons.
6. Figures 1 and 2 are informative but could be improved through clearer labeling and reporting of confidence intervals where appropriate.
7. Data availability is restricted due to ethical considerations. It would be helpful if the authors specified whether de-identified data or aggregated datasets could be shared for replication purposes under controlled conditions.

Overall Assessment

 This manuscript contributes valuable information regarding psychosocial determinants of crowd behavior during Hajj and provides an interesting framework for integrating psychological, social, and risk perception factors into crowd management research. However, several methodological and interpretive issues require clarification, particularly regarding sampling, causal language, scale development, and model interpretation.

 Approval Status: Approved with Reservations.

Is the work clearly and accurately presented and does it cite the current literature?

Partly

If applicable, is the statistical analysis and its interpretation appropriate?

Partly

Are all the source data underlying the results available to ensure full reproducibility?

No

Is the study design appropriate and is the work technically sound?

Partly

Are the conclusions drawn adequately supported by the results?

Partly

Are sufficient details of methods and analysis provided to allow replication by others?

Partly

Reviewer Expertise:

Hajj and Mass Gatherings Research; Crowd Behavior and Crowd Safety; Public Health; Risk Perception and Risk Communication; Behavioral and Social Determinants of Health; Emergency Medical Services; Disaster Preparedness and Mass Gathering Management.

I confirm that I have read this submission and believe that I have an appropriate level of expertise to confirm that it is of an acceptable scientific standard, however I have significant reservations, as outlined above.
